# Regulation of a High-Iron Diet on Lipid Metabolism and Gut Microbiota in Mice

**DOI:** 10.3390/ani12162063

**Published:** 2022-08-13

**Authors:** Qingqing Xiong, Jing Zhao, Chenying Tian, Wan Ma, Linfeng Miao, Li Liang, Kang Zhang, Huahua Du

**Affiliations:** Key Laboratory of Animal Feed and Nutrition of Zhejiang Province, College of Animal Sciences, Zhejiang University, Hangzhou 310058, China

**Keywords:** high-iron diet, lipid metabolism, adipogenesis, lipolysis, gut microbiota

## Abstract

**Simple Summary:**

Iron is an essential micronutrient involved in many important physiological activities and plays a key role in growth and development of animals. However, exposure to high levels of dietary iron has an effect on growth performance, lipid metabolism, and gut microbiota in animals. In this study, we evaluated the effects of a high-iron diet on lipid metabolism and gut microbiota in mice. Our study shows that a high-iron diet decreased fat accumulation and lipid deposition by downregulating adipogenesis and upregulating lipolysis in the liver and adipose tissues of mice. In addition, a high-iron diet induced slight intestinal damage with duodenal inflammation, and reshaped the gut microbiota, but the role of gut microbiota in regulating lipid metabolism needs to be further explored. Hence, this study shows that a high-iron diet can negatively affect mice health, and it is recommended to strictly control the intake of iron in livestock and poultry.

**Abstract:**

Iron homeostasis disorder is associated with the imbalance of lipid metabolism, while the specific interaction remains unclear. In the present study, we investigated the effect of a high-iron diet on lipid metabolism in mice. The C57BL/6 mice were fed with a normal diet (WT) or a high-iron diet (WT + Fe) for 12 weeks. We found that mice in the WT + Fe group showed a significant decrease in body weight gain, body fat and lipid accumulation of liver when compared with mice in the WT group. Accordingly, serum total cholesterol and triglyceride levels were both reduced in mice with a high-iron diet. Moreover, mice in the WT + Fe group exhibited a significant decrease in expression of genes regulating adipogenesis and adipocyte differentiation, and a significant increase in expression of fat hydrolysis enzyme genes in both liver and adipose tissues, which was consistent with their dramatic reduction in adipocyte cell size. In addition, a high-iron diet decreased the relative abundance of beneficial bacteria (*Akkermansia*, *Bifidobacterium* and *Lactobacillus*) and increased the relative abundance of pathogenic bacteria (*Romboutsia* and *Erysipelatoclostridium*). Thus, our research revealed that a high-iron diet reduced lipid deposition by inhibiting adipogenesis and promoting lipolysis. Altered gut microbial composition induced by a high-iron diet may not play a critical role in regulating lipid metabolism, but might cause unwanted side effects such as intestinal inflammation and damaged villi morphology at the intestinal host–microbe interface. These findings provide new insights into the relationship among iron, lipid metabolism and gut microbiota.

## 1. Introduction

Iron is an essential micronutrient that plays a key role in growth and development of mammals. Iron is involved in numerous physiological processes, including synthesis of heme [1], maintenance of normal immune function [2], oxygen transport and synthesis of metabolic enzymes [3]. In addition, recent studies have highlighted the important role of iron in the balance of lipid homeostasis. For example, in a human cohort, the distribution of iron in adipose tissue was negatively correlated with body mass index [4]. Animal studies have shown that body weight, liver weight, and fat weight are also reduced after iron treatment in a high-fat mouse model [5]. Thus, the question is what changes in lipid metabolism result from dietary iron changes.

Scientific evidence suggests an unexpected relationship between iron and the regulation of lipid metabolism. In the model of insulin resistance in visceral adipose tissue induced by dietary iron overload, iron-overloaded mice showed lower visceral adipose tissue mass in the epididymal fat pad, which was associated with iron accumulation in adipocytes [6]. Our previous study also showed that dietary excess iron significantly decreased body weight and lipid droplets in the liver, and triglyceride content also decreased by 27% in iron overload *db/db* mice [7]. Although the factors that affect lipid metabolism by a high-iron diet are still uncertain, it is increasingly recognized that dietary iron affects the gut microbiota. Human growth and health are positively correlated with the consumption of iron and are highly affected by the gut microbiota composition [8]. Only 10% of dietary iron is absorbed by the body, and the remaining 90% is excreted through feces, thus affecting the microbiota balance [9]. Iron is required for the growth and virulence of many pathogenic gut bacteria [10]. Deficiencies and excesses of iron levels can contribute to gut dysbiosis and the progression of inflammation and colorectal cancer [11]. According to a study, iron fortification had an effect on the gut microbiota in weaning African infants, decreasing the abundance of *Bifidobacteria*, and increasing the abundance of some specific enteropathogens such as pathogenic *Escherichia coli* [12]. There is also a significant interplay among gut microbiome, iron status, and hepatic fat accumulation, with potential implications for targeted therapy of NAFLD [13].

In livestock and poultry production, farms typically inject iron dextran as iron supplementation to prevent piglets from slow growth and development due to iron deficiency. In addition, most feed raw materials already contain high level of iron, which easily leads to a higher iron supplementation of pigs than their demand, and then affects the normal growth and development of the body. It has been found that piglets fed an excessive-iron diet have an increased risk of diarrhea [14,15]. In the case of infection, iron supplementation will reduce the growth performance of mice, aggravate bacterial infection, and even lead to death [16]. Moreover, the nutritional role of iron is often focused on the growth, development and production performance of livestock and poultry, but ignoring the impact of iron on lipid metabolism and gut microbiota.

Studies have shown that dietary iron affects gut microbiota and lipid metabolism. However, it remains unclear whether a high-iron diet has an effect on lipid metabolism through the gut microbiota. Therefore, this study hypothesized that a high-iron diet directly regulates lipid metabolism through gut microbiota. To investigate how iron changes lipid metabolism in mice by studying the effects of a high-iron diet on body weight, liver and fat metabolism, and gut microbiota, aiming to provide some evidence for iron changing lipid metabolism and provide new insights into the use of iron on livestock and poultry.

## 2. Materials and Methods

### 2.1. Animals

A total of 12 male C57BL/6 mice (6 weeks old, GemPharmatech Co., Ltd., Nanjing, China) were pre-fed for 1 week in the Experimental Animal Center of Zhejiang University. They were randomly divided into two groups (*n* = 6 per group). These mice were provided with a normal diet (45 mg Fe/kg food, WT group) or a high-iron diet (normal diet plus ferrous sulfate, 1250 mg Fe/kg food, WT + Fe group) for 12 weeks, based on the AIN-93G formulation. Mice were housed under standard temperature conditions (22 ± 1 °C) with a regular 12 h light/dark cycle and water and food *ad libitum*. The body weight of the mice was recorded every 4 days. At the 12th week, all mice were sacrificed. Serum, liver, duodenum, jejunum and adipose tissue samples of mice were collected, quickly placed in liquid nitrogen and stored at −80 °C. All animal procedures were approved by Animal Ethics Committee of Zhejiang University (approval no. 20077).

### 2.2. Serum Biochemical Assays

Mice serum was separated from blood by centrifugation at 3000× *g* for 15 min (6 mice per group). The upper serum was carefully collected with a pipette into a centrifuge tube and stored at −80 °C before testing. Serum blood glucose, total triglyceride, total cholesterol, alanine aminotransferase (ALT), aspartate aminotransferase (AST) and iron level were detected using kits (Jiancheng Biology, Nanjing, China) according to the manufacturer’s instructions, using a glucose kit, a triglyceride assay kit, a total cholesterol assay kit, an alanine aminotransferase assay kit, an aspartate aminotransferase assay kit and a serum iron assay kit. Serum insulin level was quantified using a murine ELISA kit (Jiancheng Biology, Nanjing, China).

### 2.3. Histological Analysis

Three mice per group were randomly used for tissue quantification, then more than three tissue quantification images were taken from each mouse under different microscope multiples, and finally these images were quantitatively analyzed. The specific steps are as follows. Liver, duodenum, jejunum, and adipose tissue sections were fixed with 4% paraformaldehyde for 24 h, dehydrated sequentially with graded ethanol, and embedded in paraffin. Sections of 5 µm were deparaffinized and rehydrated. Nuclei and cytoplasm were stained in hematoxylin and eosin solution, and finally use graded alcohol for dehydration and mounting. Liver section was fixed with 4% paraformaldehyde at 4 °C for 24 h, rinsed with PBS, embedded, and quickly frozen in −80 °C pre-cooled dimethylbutane. Sections of 10 µm were stained in Oil Red O solution for 40 min, eluted with 40% ethanol for 2 min, counterstained with hematoxylin for 1 min, rinsed with water for 2 min, dried and mounted with glycerol. Image-Pro Plus was used to calculate the lipid droplet area by Oil Red O staining. The morphology of duodenal microvilli was detected by scanning electron microscopy (SEM). Duodenum section was fixed with 2.5% glutaraldehyde at 4 °C overnight, rinsed three times with 0.1 M phosphate buffer, pH 7.0, fixed with 1% osmic acid for 1–2 h. After rinsing the sample three times with phosphate buffer, the section was dehydrated with graded ethanol, and finally subjected to critical point drying and coating. Duodenum samples were visualized by Hitachi SU8010 FASEM (Tokyo, Japan).

### 2.4. RNA Extraction and Quantitative Real-Time PCR

Total RNA was isolated from liver, duodenum and adipose tissue using Trizol^®^ (Biosharp, Hefei, China) and cDNAs were synthesized using Hifair^®^ III 1^st^ Strand cDNA Synthesis SuperMix for qPCR (Yeasen Biotechnology, Shanghai, China) according to the manufacturer’s instructions (6 mice per group). Real-time PCR was performed using Hief UNICON^®^ qPCR SYBR Green Master Mix (Yeasen Biotechnology, Shanghai, China) and the ABI 7500 real-time PCR system (Thermo Fisher Scientific, Waltham, MA, USA). The primer sequences are listed in Table 1. GAPDH is the reference gene.

### 2.5. Western Blot Analysis

Total protein was isolated from liver and adipose tissues (6 mice per group) using PMSF and cell lysis buffer for Western and IP (KeyGen Biotech, Nanjing, China). The total protein concentration was determined using the BCA Protein Assay Kit (KeyGen Biotech, Nanjing, China) according to the manufacturer’s instructions. Protein samples (20 µg) were separated by 8% to 15% sodium dodecyl sulfate-polyacrylamide gel electrophoresis (SDS-PAGE) at 80 V for 30 min and at 110 V for 1 h, and then transferred onto a polyvinylidene difluoride (PVDF) membrane (Sigma-Aldrich, St. Louis, MO, USA) at 260 mA for 1 h. The membrane was blocked with skimmed milk for 1 h at room temperature and washed three times with TBST for 10 min each time. Then, the membrane was incubated with primary antibodies overnight at 4 °C, including β-actin (1:5000, Abcam, Cambridge, UK), HSL (1:1000, Cell Signaling Technology, Danvers, MA, USA), ACC (1:1000, Cell Signaling Technology, Danvers, MA, USA), FAS (1:1000, Cell Signaling Technology, Danvers, MA, USA), C/EBPα (1:1000, Cell Signaling Technology, Danvers, MA, USA), CD36 (1:1000, Cell Signaling Technology, Danvers, MA, USA), ATGL (1:1000, HuaBio, Hangzhou, China). Subsequently, the membrane was washed three times with TBST, and incubated with a HRP-conjugated secondary antibodies (1:10,000, Biosharp, Hefei, China) for 1 h at room temperature. Finally, target proteins were visualized using ECL Detection Kit (KeyGen Biotech, Nanjing, China), and analyzed using ImageJ software.

### 2.6. Gut Microbiota Analysis

Total microbial genomic DNA was extracted from cecum in two groups (5 mice per group). DNA concentration and purity were monitored on 1% agarose gels. The V3–V4 gene regions of the bacterial 16S rRNA gene were amplified with forward primer (338F-ACTCCTACGGGAGGCAGCAG) and reverse primer (806R-GGACTACHVGGGTWTCTAAT). The MiSeq platform was used to describe the bacterial community, including OUT clustered analysis, species annotation, sample complexity analysis, multi-sample comparative analysis, significant analysis of community structure differences between groups, and Kyoto Encyclopedia of Genes and Genomes (KEGG) functional prediction analysis.

### 2.7. Statistical Analysis

All assays were performed at least in triplicate and the values were presented as the mean ± SEM. Statistical analysis were performed using GraphPad Prism 8.0 and group differences were compared by an unpaired two-tailed Student’s *t* test. Values of *p* < 0.05 were considered statistically significant. Statistical significance is displayed as * *p* < 0.05 or ** *p* < 0.01.

## 3. Result

### 3.1. A High-Iron Diet Reduced the Body Weight and Changed Iron Metabolism of Mice

**Figure 1:**
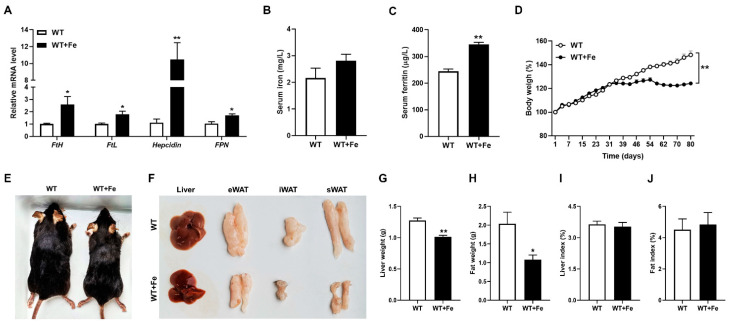
A high-iron diet reduced the body weight and changed iron metabolism of mice. (**A**) *Hepcidin*, *FtH*, *FtL* and *FPN* mRNA levels in liver. (**B**) Serum iron concentration. (**C**) Serum ferritin concentration. (**D**) Body weight. (**E**) Representative images of body size of mice. (**F**) Representative images of liver, eWAT, iWAT, and sWAT. (**G**) Liver weight. (**H**) Fat weight. (**I**) Liver index. (**J**) Fat index. * *p* < 0.05, ** *p* < 0.01.

We first evaluated the effects of a high-iron diet on iron metabolism and growth performance of mice. As expected, our results showed that the mRNA expression of *Hepcidin* (*p* < 0.01), *FtH* (*p* < 0.05), *FtL* (*p* < 0.05) and *FPN* (*p* < 0.05) were significantly increased in WT + Fe mice (Figure 1A). There was no difference in serum iron concentration of mice between the two groups (Figure 1B), while serum ferritin level was significantly (*p* < 0.01) higher in WT + Fe mice (Figure 1C). These results indicated that a high-iron diet changed iron metabolism of mice.

Growth data showed that the body weight of the two groups has a significant difference on the 39th day (*p* < 0.01), the body weight in WT + Fe mice stabilized from the fifth week, while the body weight in WT mice kept increasing. At the end of the trial, weight gain in WT mice was 20 percent, while weight gain in WT + Fe mice was 40 percent. That is to say, the body weight in WT + Fe mice reduced significantly (Figure 1D). The appearance of mice also intuitively reflected the smaller size of WT + Fe mice (Figure 1E). Moreover, we also noticed that the size and weight of liver (*p* < 0.01) and adipose tissues (*p* < 0.05), including epididymal white adipose tissue (eWAT), inguinal white adipose tissue (iWAT) and subcutaneous white adipose tissue (sWAT) were significantly reduced (Figure 1F–H) in WT + Fe mice. These data further proved that a high-iron diet could reduce the body weight gain of mice. There were no significant differences in liver index (liver weight/body weight) (Figure 1I) and fat index (fat weight/body weight) (Figure 1J) between the two groups.

### 3.2. A High-Iron Diet Inhibited Adipogenesis and Promoted Lipolysis in Liver

**Figure 2:**
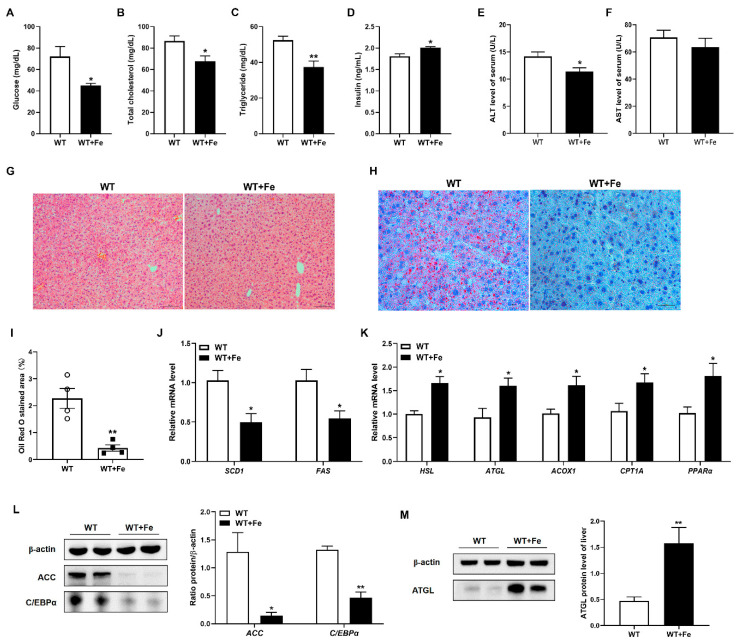
A high-iron diet inhibited adipogenesis and promoted lipolysis in liver. (**A**) Serum glucose. (**B**) Serum total cholesterol. (**C**) Serum triglycerides. (**D**) Serum insulin. (**E**) Serum AST. (**F**) Serum AST. (**G**) H & E staining of liver. (**H**) Oil Red O staining of liver. (**I**) Statistical area of lipid droplets (*n* = 4). (**J**) *SCD1* and *FAS* mRNA levels in liver. (**K**) *HSL*, *ATGL*, *ACOX1*, *CPT1A* and *PPARα* mRNA levels in liver. (**L**) *ACC* and *C/EBPα* protein levels in liver. (**M**) *ATGL* protein levels in liver. Data are presented as the mean ± SEM. * *p* < 0.05, ** *p* < 0.01. Original western blot figures in Figure S1.

To better understand the observed reduction in fat deposits in WT + Fe mice, we examined serum biochemical parameters associated with lipid metabolism and hepatic fat deposition. The concentration of serum glucose (*p* < 0.05), total cholesterol (*p* < 0.05) and triglyceride (*p* < 0.01) were significantly decreased in WT + Fe mice (Figure 2A–C), insulin level was significantly (*p* < 0.05) increased (Figure 2D). Moreover, serum ALT (*p* < 0.05) and AST level reduced in WT + Fe mice (Figure 2E,F), which indicated that the liver of mice was not damaged after feeding with a high-iron diet. H&E staining showed that there was no difference in hepatocyte morphology of mice between the two groups, and no pathological changes were observed (Figure 2G).

Oil red O staining of liver section showed that lipid accumulation reduced significantly in WT + Fe mice compared with WT mice (Figure 2H,I). Meanwhile, a high-iron diet significantly (*p* < 0.05) decreased the transcripts of adipogenesis-related enzyme genes such as stearoyl-CoA desaturase 1 (*SCD1*) and fatty acid synthase (*FAS*) (Figure 2J), while increased the mRNA expression of fat hydrolysis enzyme genes such as hormone sensitive lipase (*HSL*) and adipose triglyceride lipase (*ATGL*), and fatty acid oxidation markers including acyl-CoA oxidase 1 (*ACOX1*), carnitine palmitoyltransferase 1A (*CPT1A*), and peroxisome proliferator-activated receptor α (*PPARα*) in the liver of mice (Figure 2K). Western blot analysis showed that a high-iron diet dramatically inhibited the protein expression of lipid metabolism regulators, including acetyl-CoA carboxylase (*ACC*) and CCAAT/enhancer binding protein α (*C/EBPα*) in liver. Compared with the WT group, the expression of *ACC* and *C/EBPα* decreased 89% and 65% in WT + Fe mice (Figure 2L). Meanwhile, the protein expression of *ATGL* promoted 3.34 folds after feeding with a high-iron diet (Figure 2M). These results indicated that a high-iron diet could reduce lipid accumulation by inhibiting adipogenesis and promoting lipolysis without pathological damage in liver.

### 3.3. A High-Iron Diet Inhibited Adipogenesis and Promoted Lipolysis in Adipose Tissues

**Figure 3:**
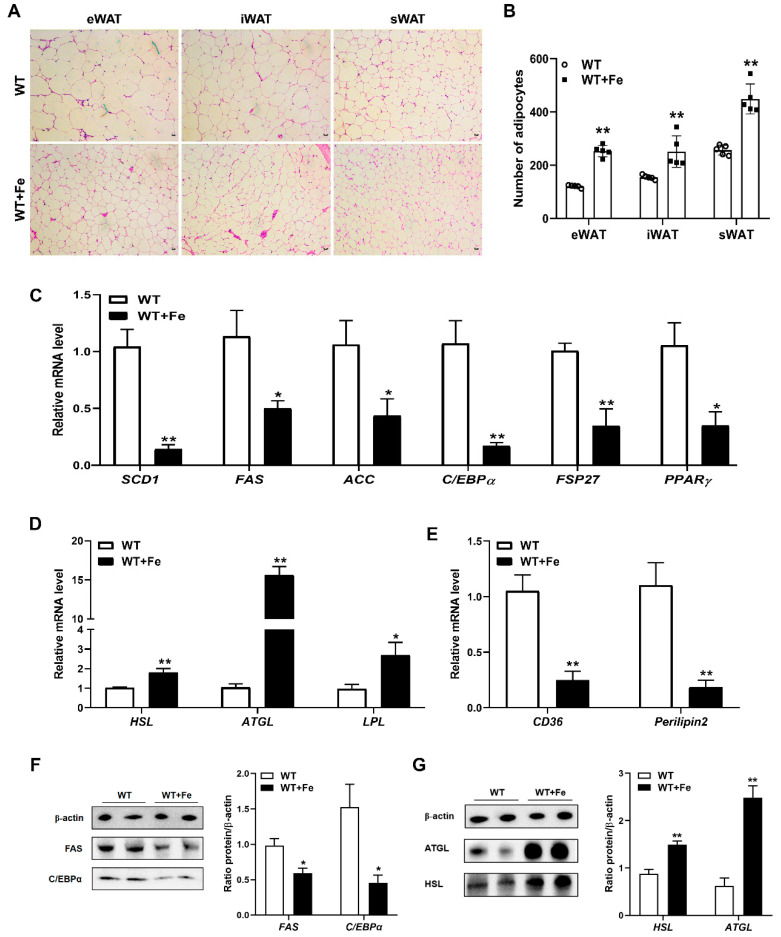
A high-iron diet inhibited adipogenesis and promoted lipolysis in adipose tissues. (**A**) H&E staining of eWAT, iWAT, and sWAT. (**B**) Statistical number of adipocytes (*n* = 5). (**C**) *SCD1*, *FAS*, *ACC*, *C/EBPα*, *FSP27* and *PPARγ* mRNA levels in adipose tissue. (**D**) *HSL*, *ATGL* and *LPL* mRNA levels in adipose tissue. (**E**) *CD36* and *Perilipin2* mRNA levels in adipose tissue. (**F**) *FAS* and *C/EBPα* protein levels in adipose tissue. (**G**) *HSL* and *ATGL* protein levels in adipose tissue. Data are presented as the mean ± SEM. * *p* < 0.05, ** *p* < 0.01. Original western blot figures in Figure S1.

High iron-induced weight loss may be related to the metabolism of adipose tissue in mice. H&E staining showed that the size of adipocytes was smaller in all detected eWAT, iWAT and sWAT of WT + Fe mice (Figure 3A), and the number of adipocytes was all significantly (*p <* 0.01) increased in the same visual field (Figure 3B). Moreover, the mRNA expression of genes regulating adipogenesis, such as *SCD1* (*p* < 0.01), *FAS* (*p* < 0.05) and *ACC* (*p* < 0.05) was decreased significantly in adipose tissues of mice after feeding with a high-iron diet, and the mRNA expression of genes regulating adipocyte differentiation, including *C/EBPα* (*p* < 0.01), fat-specific protein of 27 kD (*FSP27*) (*p* < 0.01) and peroxisome proliferator-activated receptor γ (*PPARγ*) (*p* < 0.05) was also decreased significantly (Figure 3C). However, the mRNA expression of fat hydrolysis enzyme genes such as *HSL* (*p* < 0.01), *ATGL* (*p* < 0.01) and lipoprotein lipase (*LPL*) (*p* < 0.05) was significantly increased (Figure 3D). In addition, the mRNA expression of cluster of differentiation 36 (*CD36*) (*p* < 0.01) and *Perilipin2*, as lipid droplet marker, (*p* < 0.01) was significantly decreased (Figure 3E). Western blot analysis showed that the protein expression of *FAS* and *C/EBPα* in adipose tissues was significantly decreased by 40% and 70% of WT + Fe mice (Figure 3F). Moreover, the protein expression of *ATGL* and *HSL* was significantly increased by 4.0 times and 1.7 times, respectively, after feeding with a high-iron diet (Figure 3G). These results indicated that a high-iron diet could reduce the size of adipocytes, inhibit adipogenesis and promote lipolysis in adipose tissues of mice.

### 3.4. A High-Iron Diet Reshaped the Gut Microbiota of Mice

**Figure 4:**
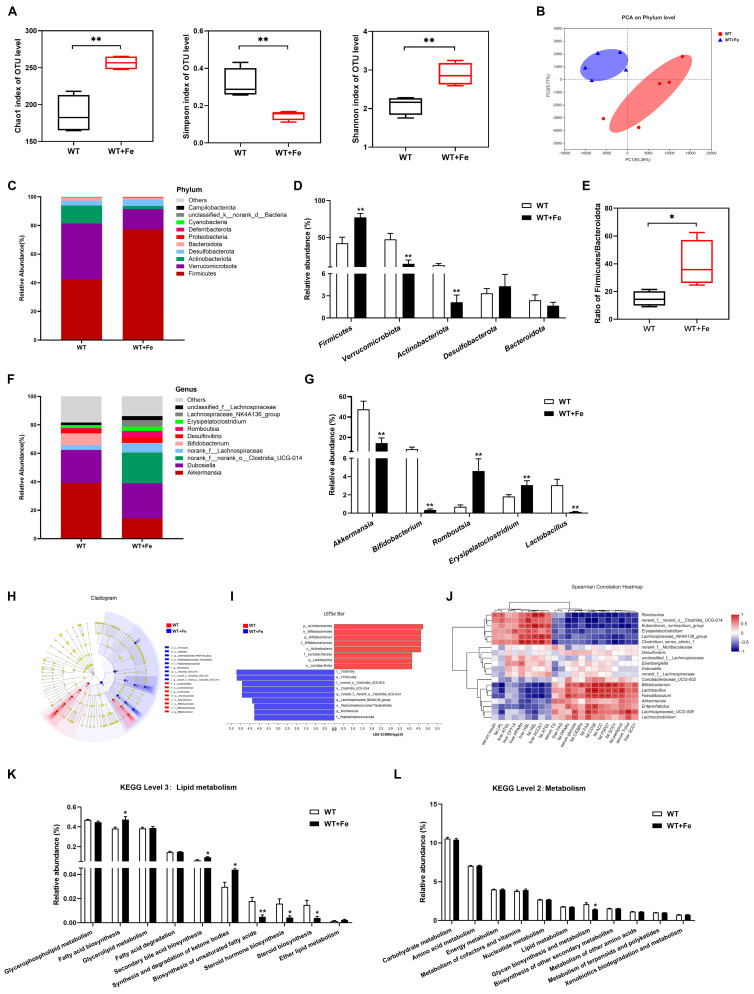
A high-iron diet reshaped the gut microbiota of mice. (**A**) Alpha diversity. (**B**) PCA plot. (**C**) Microbiota distribution at the phylum level. (**D**) Relative abundance of dominated phylum. (**E**) Ratio of Firmicutes/Bacteroidetes. (**F**) Microbiota distribution at the genus level. (**G**) Relative abundance of dominated genus. (**H**) Cladograms. (**I**) LEfSe analysis, LDA scores > 4.0. (**J**) Spearman correlation heatmap at the top 20 genus level. (**K**) Microbial metabolic function of KEGG pathways at level 3 lipid metabolism. (**L**) Microbial metabolic function of KEGG pathways at level 2 metabolism. Data are presented as the mean ± SEM. * *p* < 0.05, ** *p* < 0.01.

The results showed that a high-iron diet reshaped the composition of gut microbiota. Alpha diversity analysis of gut microbiota revealed that Chao1 (*p* < 0.01) and Shannon (*p* < 0.01) indexes were higher, and Simpson index was lower (*p* < 0.01) in WT + Fe mice (Figure 4A), indicating a high-iron diet improved the richness and diversity of gut microbiota. Beta-diversity was estimated by principal component analysis (PCA). The two groups clustered separately, unveiling that there were differences in the composition of gut microbiota between two groups (Figure 4B).

The phylum Firmicutes and Verrucomicrobiota were the most two prevalent taxa in both two groups, followed by Actinobacteriota (Figure 4C). Compared to the WT group, the relative abundance of Firmicutes was increased by 45.3% (*p* < 0.01), but Verrucomicrobiota and Actinobacteriota were decreased (*p* < 0.01) by 69.9% and 82.8% in WT + Fe mice (Figure 4D), and the ratio of Firmicutes/Bacteroidetes was increased by 2.68 times (Figure 4E). Iron treatment also changed the composition of gut microbiota at genus level (Figure 4F). The relative abundances of beneficial bacteria including *Akkermansia*, *Bifidobacterium* and *Lactobacillus* were decreased (*p* < 0.01) by 69.9%, 95.9% and 95.4%, while those of pathogenic bacteria such as *Romboutsia* and *Erysipelatoclostridium* were increased significantly (*p* < 0.01) in WT + Fe mice (Figure 4G). Cladograms and LEfSe analysis showed that *Romboutsia* of phylum Firmicutes were key bacterium in the WT + Fe group, while the key bacteria in the WT group were mainly *Lactobacillus* of phylum Firmicutes and *Bifidobacterium* of phylum Actinobacteriota (Figure 4H,I).

To further clarify the possible relationship between the lipid metabolism and gut microbiota altered after feeding with a high-iron diet, the heat map shows correlation through Spearman’s calculation at the top 20 genus level. As shown in Figure 4J, the genes related to promote adipogenesis, including *PPARγ, FAS*, *SCD1*, *ACC*, *FSP27*, *CD36* and *Perilipin2*, were positively correlated with *Bifidobacterium*, *Lactobacillus* and *Faecalibaculum*, but negatively correlated with *Romboutsia*, *norank_f__norank_o__Clostridia_UCG-014* and *Eubacterium_ruminantium_group*. The genes related to lipolysis, such as *HSL, ATGL* and *ACOX1*, were positively correlated with *Romboutsia*, *Eubacterium_ruminantium_group* and *Lachnospiraceae_NK4A136_group*, but negatively correlated with *Bifidobacterium*, *Lactobacillus* and *Faecalibaculum*. Moreover, the metabolic pathways are compared by Kyoto Encyclopedia of Genes and Genomes (KEGG) functional prediction analysis. The relative abundances of fatty acid biosynthesis, secondary bile acid biosynthesis, and synthesis and degradation of ketone bodies were significantly (*p* < 0.05) increased in the WT + Fe group (Figure 4K). However, the relative abundances of biosynthesis of unsaturated fatty acids (*p* < 0.01), steroid hormone biosynthesis (*p* < 0.05) and steroid biosynthesis (*p* < 0.05) were significantly decreased in the WT + Fe group (Figure 4K). Notably, there was no difference in the relative abundance of lipid metabolism between the two groups at level 2 metabolism (Figure 4L), which suggested that reshaped bacteria induced by a high-iron diet may not play a key role in lipid metabolism.

### 3.5. A High-Iron Diet Induced Intestinal Damage of Mice

**Figure 5:**
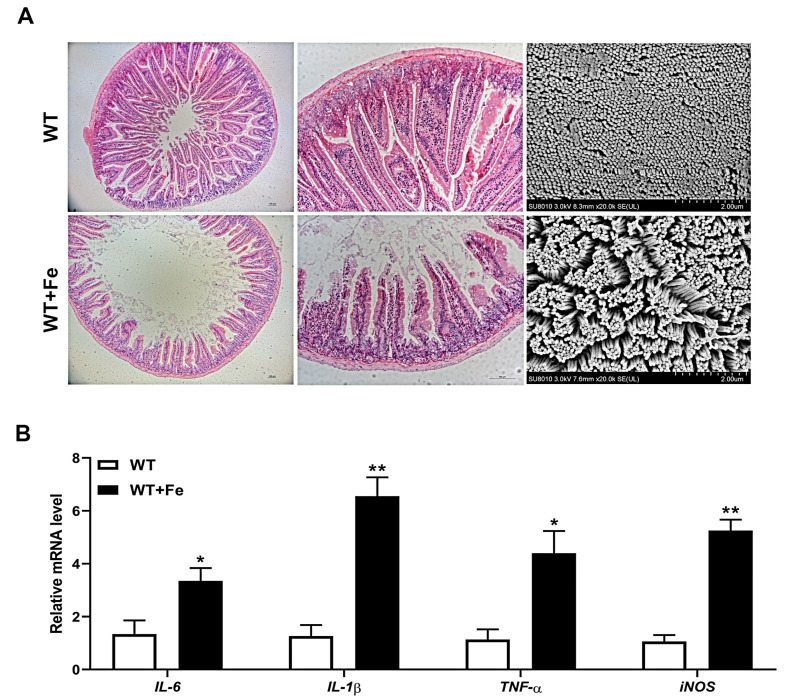
A high-iron diet induced intestinal damage of mice. (**A**) H&E staining and SEM of the duodenum. (**B**) *IL-6*, *IL-1β*, *TNF-α* and *iNOS* mRNA levels in the duodenum. Data are presented as the mean ± SEM. * *p* < 0.05, ** *p* < 0.01.

H&E staining showed that a high-iron diet caused slight damage of the duodenum with shortened and disarrayed villi, and SEM observation also showed that the arrangement of duodenal microvilli was obviously sparse in the WT + Fe group compared with the WT group (Figure 5A). In addition, RT-qPCR analysis showed that the transcripts of proinflammatory cytokines including *IL-6* (*p* < 0.05), *IL-1β* (*p* < 0.01), *TNF-α* (*p* < 0.05) and *iNOS* (*p* < 0.01) were all significantly increased in the duodenum of mice after feeding with a high-iron diet (Figure 5B). The results suggested that a high-iron diet induced impaired intestinal villi and inflammation, which indicated that excess iron might have a negative effect on the intestinal barrier.

## 4. Discussion

Iron deficiency and obesity are global health problems that affect billions of people worldwide [17]. Growing evidence supports the existence of an association between obesity and iron deficiency [18], but the role and mechanisms of iron in the physiology of lipid metabolism remain unclear. Our results demonstrated that a high-iron diet reduced the body weight of mice, downregulated adipogenesis and upregulated lipolysis. Iron is necessary for normal physiology. However, excess iron is toxic as it accelerates the fenton reaction that produces reactive oxygen species and severely damages cells and tissues [19]. Liver is essential for iron homeostasis and lipid metabolism, thus iron overload or iron deficiency may affect lipid metabolism in the liver [20]. In our study, a 1250 mg Fe/kg diet had no adverse effect on liver function with lower ALT and AST levels, both of which are important indicators for diagnosing liver damage. When pathogenic factors lead to hepatocyte degeneration and cell membrane permeability increase, ALT is mainly released from the cells. However, when hepatocytes are seriously injured and necrotic, the AST in mitochondria is released, resulting in a significant increase in serum AST. Liver, as the largest iron storage organ, has a high tolerance to iron, and due to the low availability of oral iron, dietary iron of a 1250 mg Fe/kg diet does not cause toxicity damage to liver.

Adipose tissue is an important site for lipid storage, energy homeostasis, and whole-body insulin sensitivity, which can be regulated by the endocrine actions of various peptides and steroid hormones [21]. The number of adipocytes is basically fixed in early adulthood. Therefore, the expansion of adipose tissue mass after maturity is mainly by increasing the size of adipocytes. Studies have shown that there is a positive correlation between lipid mass and adipocyte volume in subcutaneous and visceral adipose tissue [22,23]. Another study showed that significant weight gain in non-obese adult men over several months resulted in a significant increase in adipocyte volume but no change in adipocyte number [24]. Our study showed that there was no difference in fat index but significant reduction in fat weight in mice fed a high-iron diet. Moreover, the HE staining sections showed reduced adipocyte volume after feeding with a high-iron diet in mice, suggesting a relationship with reduced body weight and adipose tissue mass.

*C/EBPα*, *FSP27* [25] and *PPARγ* [26] are the main regulators involved in adipocyte differentiation. Adipogenesis is accompanied by elevated expression of adipogenesis-related enzymes, including *FAS*, *SCD1* and *ACC* [27]. *CD36* is considered to be a lipid and fatty acid receptor for fatty acid transport [28]. *Perilipin2* is a marker for lipid droplet storage and its expression mirrors the lipid content of the cell [29]. These downregulated genes indicated that a high-iron diet would reduce adipocyte adipogenesis and differentiation. Lipolysis is the process by which Triglycerides are hydrolyzed to free fatty acids and glycerol. In adipocytes, it is achieved through sequential action of *HSL, ATGL*, and monoglyceride lipase [30]. *CPT1A*, a rate-limiting fatty acid oxidation enzyme, is responsible for fatty acids to the mitochondria for further oxidation by converting acyl-CoA to acyl-carnitine [31]. *PPARα* [32] and *ACOX1* are also an important regulator of fatty acid oxidation. These upregulated genes indicated that a high-iron diet would increase adipocyte fatty acid oxidation. Previous research also showed that the gene expression of *ACC, PPARγ and Perilipin2* was reduced in the liver of iron-overloaded mice [33]. When javelin goby *Synechogobius hasta* was exposed to waterborne iron, lipid content showed a downward trend, with downregulated *FAS, ACC,* and upregulated *HSL* [34].

The gut microbiota, an essential regulator of host metabolism, has been shown to influence lipid metabolism in mammals. Diet composition affects the composition and function of the gut microbiota, which subsequently affects host physiology. Our findings suggested that the gut microbiota was affected by a high-iron diet. Studies have shown that iron is critical for most gut bacteria [10], and Firmicutes and Bacteroidetes play important roles in regulating host lipid metabolism in the mammalian gut. A high-fat diet increased the ratio of Firmicutes/Bacteroidetes, which becomes an accepted marker of gut dysbiosis [35]. Gut microbiota of obese mice was rich in Firmicutes and Bacteroidetes decreased [36]. However, our study showed that the relative abundance of Firmicutes increased significantly and Bacteroidetes decreased after feeding with a high-iron diet. Importantly, the ratio of firmicutes/Bacteroidetes was increased. These results seem inconsistent with less fat deposition. However, it mainly proved the gut dysbiosis of mice in the WT + Fe group.

It has been reported that the abundance of *Akkermansia muciniphila* has a significant negative correlation with obesity and diabetes [37]. It could also alleviate DSS-induced acute colitis [38], which plays a key role in maintaining intestinal health [39]. The decrease in *Akkermansia* in our findings suggested that the high-iron diet may affect intestinal barrier function. *Bifidobacterium* and *Lactobacillus*, as the key bacteria in the WT group, regulate intestinal flora homeostasis. *Bifidobacteria* plays a key role in maintaining intestinal homeostasis, which is widely used as probiotics, and has beneficial effects in many pathological conditions [40]. Supplementation with *Lactobacillus plantari BNR17* could reduce visceral fat accumulation and waist circumference in obese adults [41]. Several other *Lactobacillus* have the potential to lower weight loss and fat mass in overweight subjects, including *Lactobacillus gasseri, Lactobacillus rhamnosus*, *Lactobacillus amylovorus* and *Lactobacillus plantarum* [42]. The results of these studies, which showed that weight loss in obese subjects was accompanied by an increase in beneficial bacteria, are inconsistent with the results of our study, which showed that weight loss in mice fed a high-iron diet was accompanied by a decrease in beneficial bacteria. However, the lower body weight and fat weight of WT + Fe mice in this study may be due to the regulation of adipogenesis and lipolysis at the gene level or intestinal dysfunction, rather than the direct regulation of lipid metabolism through gut microbiota. Meanwhile, our study also showed that the high-iron diet increased the relative abundance of *Romboutsia* and *Erysipelatoclostridium* in the gut microbiota of mice. *Romboutsia* and *norank_f__norank_o__Clostridia_UCG-014* was the dominant genera in the WT + Fe group. It has been reported that the abundance of *Romboutsia* and *Erysipelatoclostridium* markedly increased after acute DSS induction [43]. Notably, many bacteria from the class Clostridia have been shown to have negative effects on the gut. *Clostridium difficile*, a diarrheal pathogen, caused impaired intestinal function and elevated levels of inflammation [44]. *Clostridium perfringens* can regulation of toxin production [45]. Furthermore, KEGG analysis of gut microbiota function prediction showed that there was no significant difference in lipid metabolism between the two groups at level 2 metabolism, and there was no significant difference in fatty acid biosynthesis or decomposition at level 3 lipid metabolism.

After dietary iron enters the digestive tract, except a small part of iron that is absorbed by the small intestine, the remaining iron is absorbed into the colon along with the digesta. Iron is a key element for the growth and reproduction of most bacteria, the intestinal iron content can affect the abundance and diversity of gut microbiota. Studies showed that after dietary iron enters the gut, some Gram-negative bacteria in the gut, such as pathogenic *Escherichia coli*, *Shigella*, and *Salmonella*, begin to compete for unabsorbed iron in the gut, affecting their virulence and colonization. gut capacity [46,47]. Excess iron levels have a non-negligible role in the development of colorectal cancer [48]. In this study, the results of a high-iron diet showed that intestinal villi and microvilli were impaired and pro-inflammatory factors (*IL-6*, *IL-1β*, *TNF-α* and *iNOS*) were increased, which may be related to the decrease in beneficial bacteria (*Akkermansia, Bifidobacteria and Lactobacillus*) and the increase in pathogenic bacteria (*Romboutsia* and *Erysipelatoclostridium*).Thus, in combination with increased Firmicutes and the ratio of Firmicutes/Bacteroidetes, the present study could not provide powerful evidence to support the hypothesis that a high-iron diet can reduce fat deposition through gut microbiota directly, and it is more likely that iron first damages the intestinal barrier and then affects lipid metabolism. The specific mechanism of how a high-iron diet affects lipid metabolism remains to be further studied.

## 5. Conclusions

In conclusion, this study demonstrated that a high-iron diet decreased fat accumulation and lipid deposition by downregulating adipogenesis and upregulating lipolysis in the liver and adipose tissues of mice. A high-iron diet will undoubtedly increase luminal iron, and excess iron would affect intestinal homeostasis, including gut microbiota. As expected, a high-iron diet induced slight intestinal damage with duodenal inflammation, and reshaped the gut microbiota. However, altered microbial composition induced by a high-iron diet may not play a critical role in regulating lipid metabolism, but might cause unwanted side effects such as intestinal inflammation and damaged villi morphology at the intestinal host–microbe interface. Therefore, our research shows that a high-iron diet reduced lipid deposition by inhibiting adipogenesis and promoting lipolysis, but the role of gut microbiota in regulating lipid metabolism needs to be further explored. Subsequent in vitro experiments can be conducted to explore the specific regulatory mechanism of high iron affecting lipid metabolism using 3T3-L1 cells and other precursor adipocytes, for example, to explore how iron affects lipid metabolism in different differentiation stages of 3T3-L1 cells. In livestock and poultry production, it is recommended to strictly control the intake of iron. When weaned piglets are faced with iron-deficiency anemia, parenteral iron supplementation (intramuscular injection) should be considered instead of intestinal cavity iron supplementation (oral administration), so as to avoid intestinal inflammatory damage caused by iron and intestinal flora imbalance.

## Figures and Tables

**Table 1:** Primer sequences.

Gene	Forward Primer	Reverse Primer
ACC	CTGTATGAGAAAGGCTATGTG	AACCTGTCTGAAGAGGTTAG
ACOX1	TGTCTCGCTCCGCTCATAGG	ACATGGAGTAATTGAGGCCAACA
ATGL	ACTGAACCAACCCAACCCTTT	CCCGTCTGCTCTTTCATCCA
C/EBPα	CAAGAACAGCAACGAGTACCG	GTCACTCGTCAACTCCAGCAC
CD36	ATTCCCTTGGCAACCAACCA	CGTGGCCCGGTTCTAATTCA
CPT1A	GGGAGGAATACATCTACCTG	GAAGACGAATAGGTTTGAG
FAS	GATTCAGGGAGTGGATATTG	CATTCAGAATCGTGGCATAG
FPN	ATGGGAACTGTGGCCTTCAC	TCCAGGCATGAATACGGAGA
FSP27	ATGGACTACGCCATGAAGTCT	CGGTGCTAACACGACAGGG
FtH	TGGAACTGCACAAACTGGCTACT	ATGGATTTCACCTGTTCACTCAGATAA
FtL	CGTGGA TCTGTGTCTTGCTT	GCGAAGAGACGGTGCAGACT
GAPDH	CGGGAAACTGTGGCGTGATG	CAAAGGTGGAGGAGTGGG
Hepcidin	CTGCCTGTCTCCTGCTTCT	ATGTCTGCCCTGCTTTCTT
HSL	GGAGCTCCAGTCGGAAGAGG	GTCTTCTGCGAGTGTCACCA
IL-1β	AGTTGACGGACCCCAAAAG	TTTGAAGCTGGATGCTCTCAT
IL-6	CCCCAATTTCCAATGCTCTCC	CGCACTAGGTTTGCCGAGT
iNOS	CTCACCTACTTCCTGGACATTAC	CAATCTCTGCCTATCCGTCTC
LPL	TCGCCTTTCTCCTGATGACG	GCAATCACACGGATGGCTTC
Perilipin2	GACACCACCTGCATGGCT	TGAAGCAGGGCCACTCTC
PPARα	AGTTCGGGAACAAGACGTTG	CAGTGGGGAGAGAGGACAGA
PPARγ	GAAGCGGTGAACCACTGAT	GGAATGCGAGTGGTCTTCCA
SCD1	GTGGGGTAATTATTTGTGACC	TTTTTCCCAGACAGTACAAC
TNF-α	GCTCTTCTGTCTACTGAACTTCGG	ATGATCTGAGTGTGAGGGTCTGG

## Data Availability

Data can be obtained from the corresponding author upon reasonable request.

## Supplementary Materials

The following supporting information can be downloaded at: https://www.mdpi.com/article/10.3390/ani12162063/s1, Figure S1: Original Western Blot figures.
