# Supplementary material for: Regulation of a High-Iron Diet on Lipid Metabolism and Gut Microbiota in Mice
Source: Animals (Basel). 2022 Aug 13;12(16):2063. doi: 10.3390/ani12162063 (PMC9405328; doi:10.3390/ani12162063)

Liver

1-1 β-actin (42 kDa)

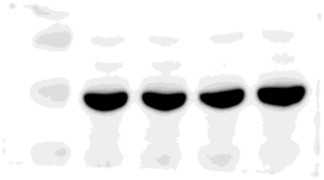

1-2 ACC (280 kDa)

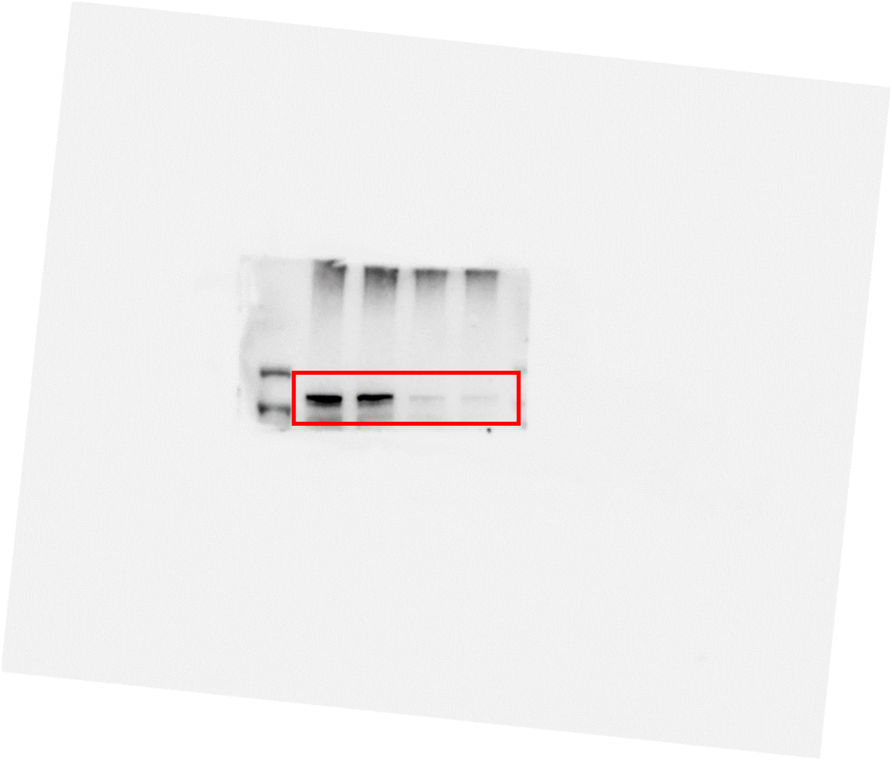

1-3 C/EBPα (42 kDa)

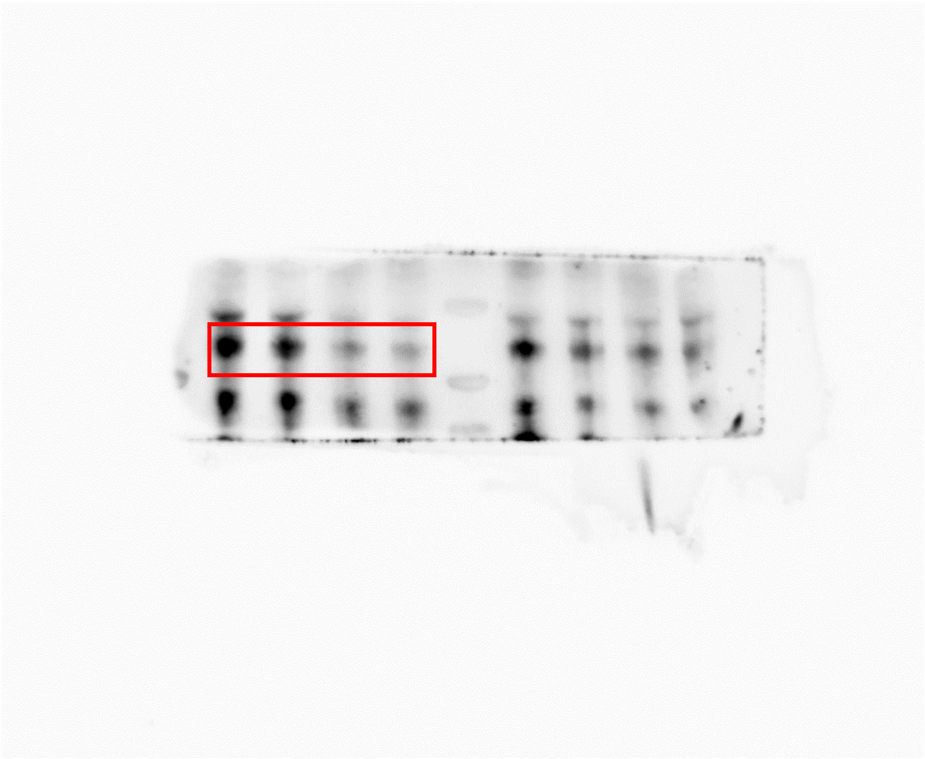

2-1 β-actin (42 kDa)

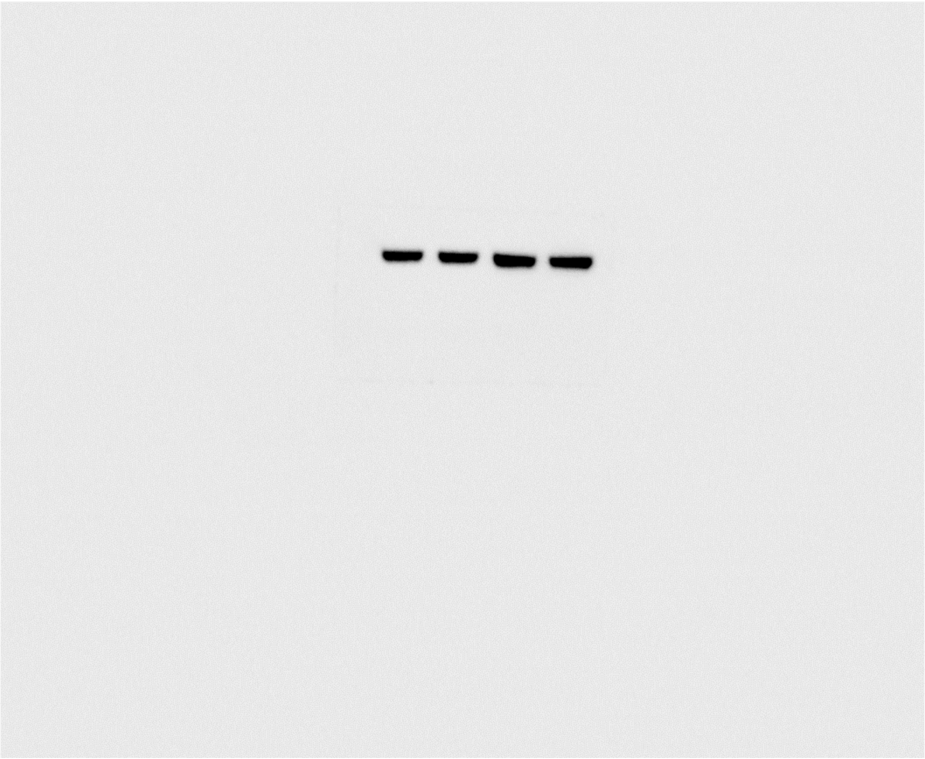

2-2 ATGL (55 kDa)

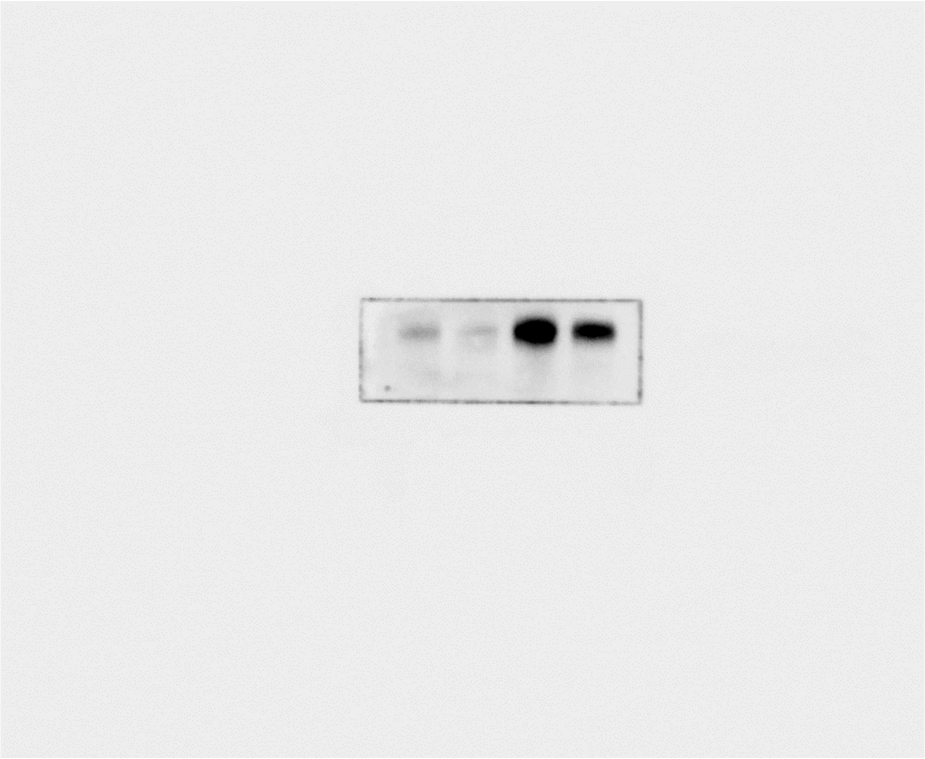

Adipose tissue

1-1 β-actin (42 kDa)

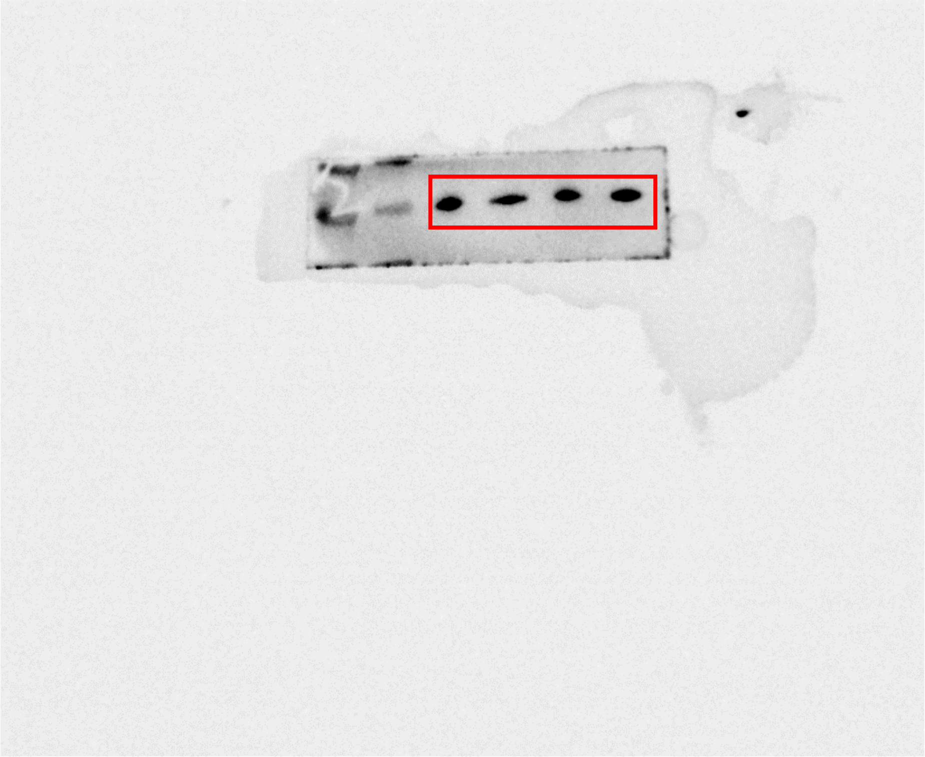

1-2 FAS (273 kDa)

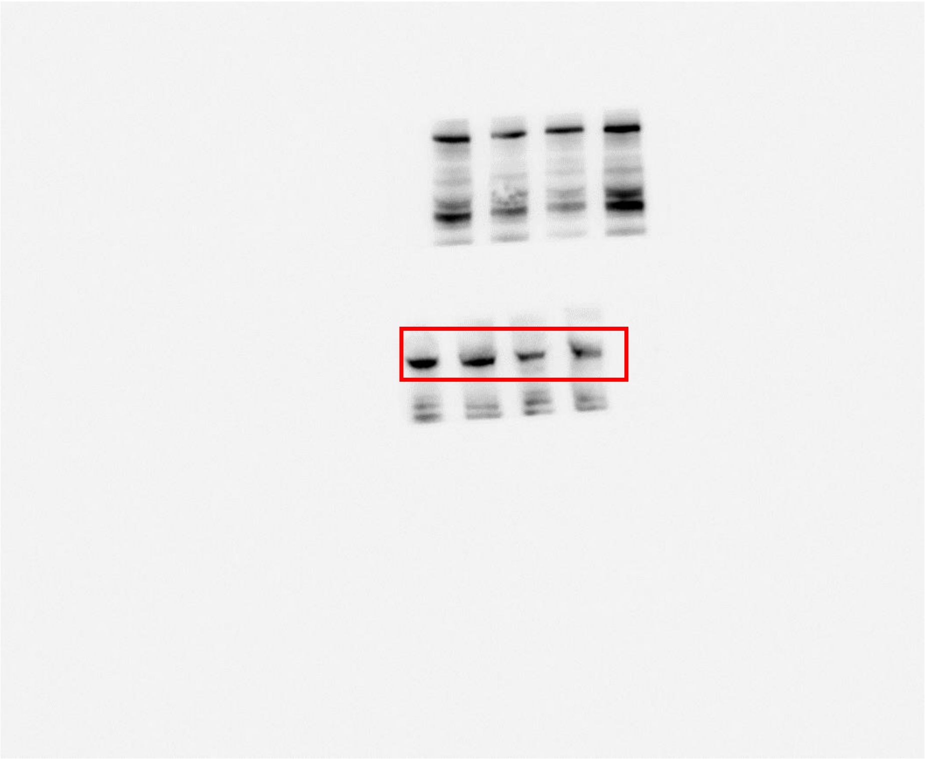

1-3 C/EBPα (42 kDa)

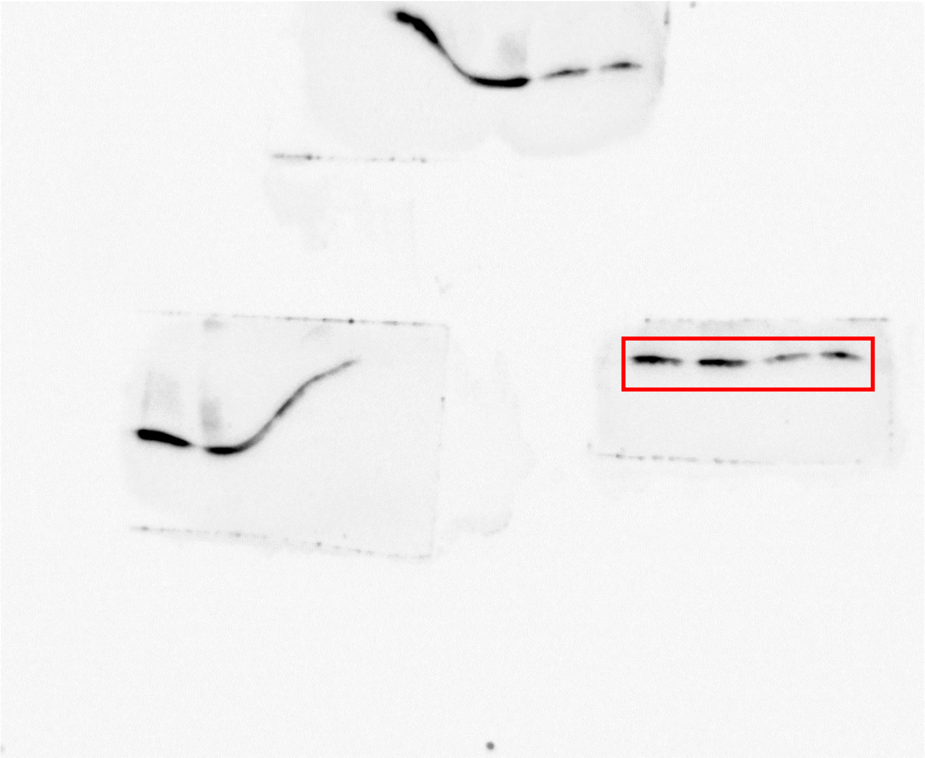

2-1 β-actin (42 kDa)

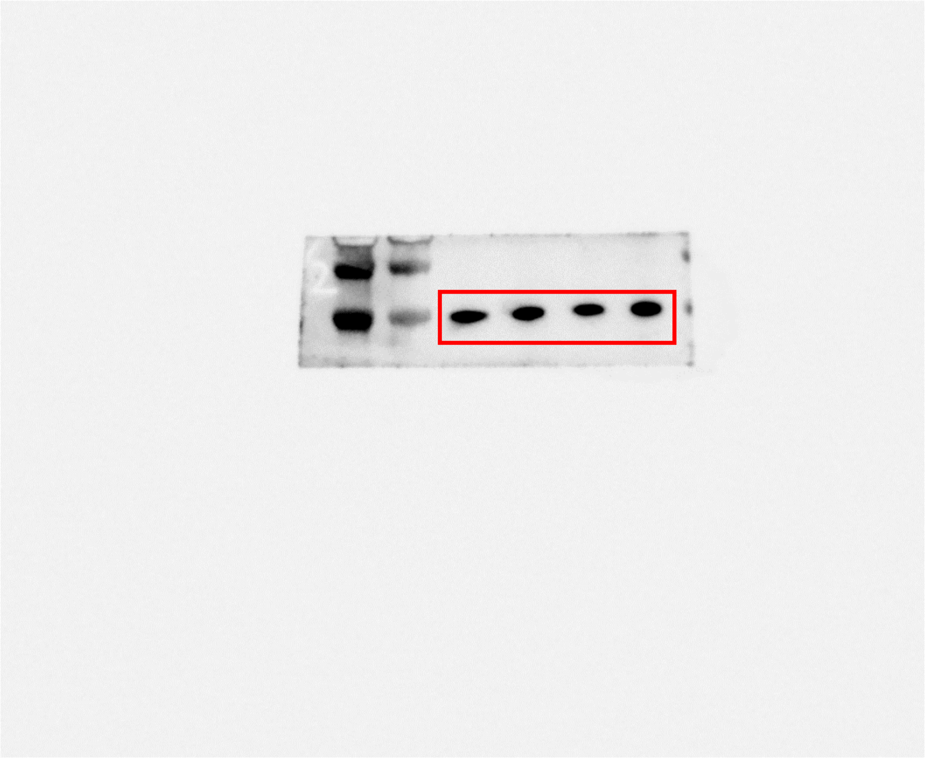

2-2 ATGL (55 kDa)

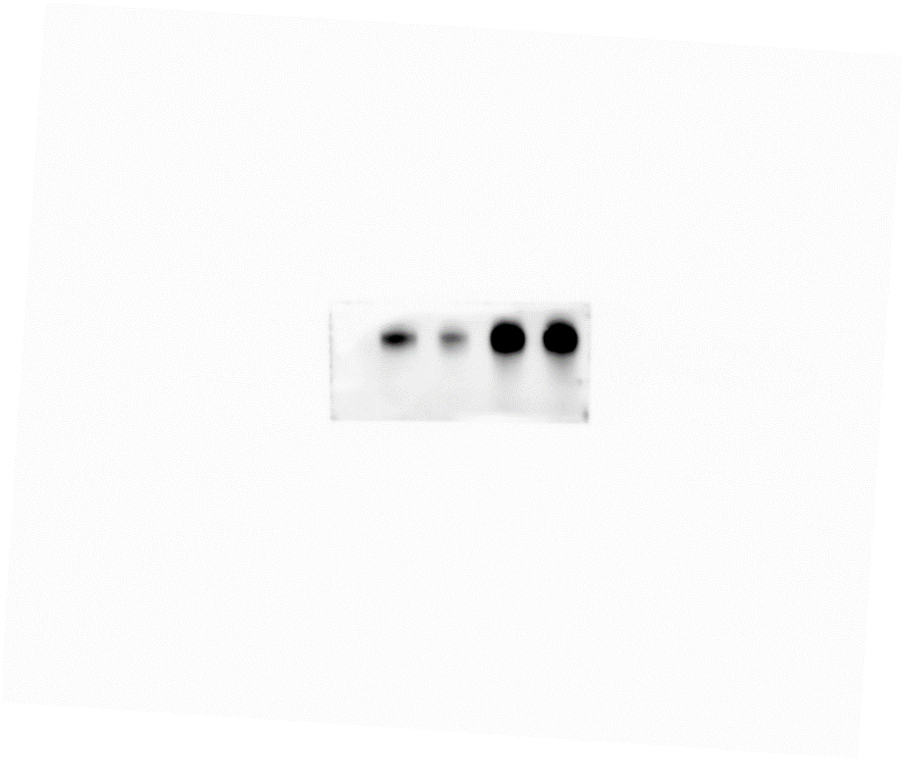

2-3 HSL (81/83 kDa)

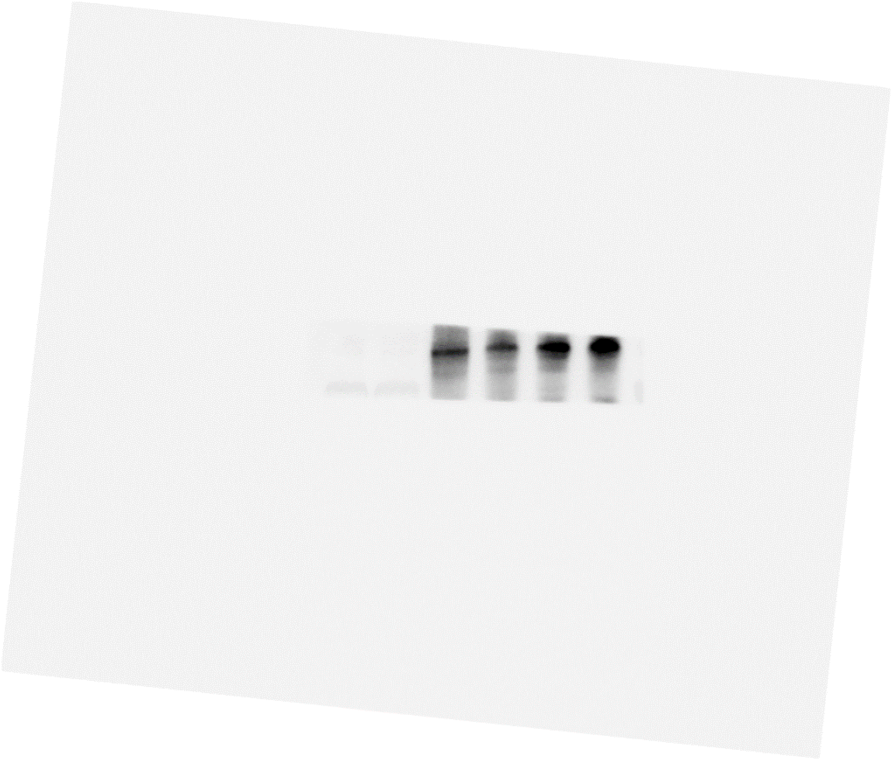

Supplement: Supplementary file 1 [file animals-12-02063-s001.zip › animals-1840361-supplementary.pdf]
